# Altered resting-state functional connectivity in emotion-processing brain regions in adults who were born very preterm

**DOI:** 10.1017/S0033291716001604

**Published:** 2016-08-15

**Authors:** C. Papini, T. P. White, A. Montagna, P. J. Brittain, S. Froudist-Walsh, J. Kroll, V. Karolis, A. Simonelli, S. C. Williams, R. M. Murray, C. Nosarti

**Affiliations:** 1 Department of Psychosis Studies, Institute of Psychiatry, Psychology and Neuroscience, King's College London, De Crespigny Park, London, UK; 2 Department of Developmental Psychology and Socialisation, University of Padua, Padua, Italy; 3 School of Psychology, University of Birmingham, Edgbaston, Birmingham, UK; 4 Division of Imaging Sciences and Biomedical Engineering, Centre for the Developing Brain, King's College London, St. Thomas’ Hospital, London, UK; 5 Department of Neuroimaging, Centre for Neuroimaging Sciences, Institute of Psychiatry, Psychology and Neuroscience, King's College London, De Crespigny Park, London, UK

**Keywords:** Emotion recognition, resting-state fMRI, very preterm birth

## Abstract

**Background:**

Very preterm birth (VPT; <32 weeks of gestation) has been associated with impairments in emotion regulation, social competence and communicative skills. However, the neuroanatomical mechanisms underlying such impairments have not been systematically studied. Here we investigated the functional integrity of the amygdala connectivity network in relation to the ability to recognize emotions from facial expressions in VPT adults.

**Method:**

Thirty-six VPT-born adults and 38 age-matched controls were scanned at rest in a 3-T MRI scanner. Resting-state functional connectivity (rs-fc) was assessed with SPM8. A seed-based analysis focusing on three amygdalar subregions (centro-medial/latero-basal/superficial) was performed. Participants’ ability to recognize emotions was assessed using dynamic stimuli of human faces expressing six emotions at different intensities with the Emotion Recognition Task (ERT).

**Results:**

VPT individuals compared to controls showed reduced rs-fc between the superficial subregion of the left amygdala, and the right posterior cingulate cortex (*p* = 0.017) and the left precuneus (*p* = 0.002). The VPT group further showed elevated rs-fc between the left superficial amygdala and the superior temporal sulcus (*p* = 0.008). Performance on the ERT showed that the VPT group was less able than controls to recognize anger at low levels of intensity. Anger scores were significantly associated with rs-fc between the superficial amygdala and the posterior cingulate cortex in controls but not in VPT individuals.

**Conclusions:**

These findings suggest that alterations in rs-fc between the amygdala, parietal and temporal cortices could represent the mechanism linking VPT birth and deficits in emotion processing.

## Introduction

Very preterm birth (VPT, <32 weeks’ gestation) has been associated with a higher risk of neurological and cognitive deficits (Bos & Roze, 2011; Burnett *et al.* 2013), behavioural problems and learning difficulties (Johnson & Marlow, 2011; Harmon *et al.* 2015). Individuals who were born VPT are vulnerable to socio-emotional impairments, including social isolation, peer rejection, poor social competence and shyness (Rickards *et al.* 2001; Dahl *et al.* 2006; Schmidt *et al.* 2008; Healy *et al.* 2013; Williamson & Jakobson, 2014), as well as impairments in the ability to recognize facial emotions (Potharst *et al.* 2013; Witt *et al.* 2014). Social deficits have been described in the first 2 years of life in VPT samples (Spittle *et al.* 2009; Boyd *et al.* 2013); and furthermore, VPT children and adolescents have been found to have a higher risk of anxiety and depression (Burnett *et al.* 2011), attention deficit hyperactivity disorder (ADHD; Lindström *et al.* 2011) and autism (Limperopoulos *et al.* 2008; Pinto-Martin *et al.* 2011) compared to controls. Individuals born VPT continue to be at an increased risk for psychiatric disorders in adult life, with mood and anxiety disorders being the most prevalent (Walshe *et al.* 2008; Nosarti *et al.* 2012; D'Onofrio *et al.* 2013). Their typical personality profile, characterized by low risk-taking, neuroticism and introversion, might also predispose them to social vulnerability and difficulties in social interactions (Allin *et al.* 2006; Saigal, 2014; Eryigit-Madzwamuse *et al.* 2015).

As VPT birth is associated with early brain injury and aberrant trajectories of cerebral development (Ball *et al.* 2012), specific structural and functional brain alterations might underlie socio-emotional impairments. Volumetric alterations have been shown in VPT samples in brain regions thought to subserve emotion processing, such as the amygdala (Peterson *et al.* 2000), orbitofrontal cortex (Giménez *et al.* 2006), fusiform gyrus (Nosarti *et al.* 2008; Gousias *et al.* 2012), hippocampus (Rogers *et al.* 2012; Omizzolo *et al.* 2013; Aanes *et al.* 2015) and insula (Nosarti *et al.* 2014). Moreover, both structural magnetic resonance imaging (MRI) and diffusion MRI studies, have found significant associations between brain alterations in regions typically implicated in socio-emotion processing and specific social and emotional outcomes in VPT samples (Rogers *et al.* 2012, 2014; Healy *et al.* 2013; Fischi-Gómez *et al.* 2015). However, to our knowledge, no neuroimaging study to date has investigated resting-state functional connectivity (rs-fc) in emotion-processing networks and performance on emotional processing tasks in individuals born VPT.

In this study, we probed the integrity of functional networks that are fundamental to emotional information processing and are anchored in the amygdala (Leppänen & Nelson, 2009; Bickart *et al.* 2014). Functional connectivity between three amygdalar subregions (centro-medial/latero-basal/superficial) and all other brain areas was investigated using rs-fc MRI. In addition, we conducted an exploratory analysis to investigate participants’ ability to recognize six basic emotions assessed with the Emotion Recognition Task (ERT; Montagne *et al.* 2007), a computer-based test that uses morphed images of human faces at different degrees of emotional intensity. In view of previous observations that amygdalar connectivity is reduced in individuals with mood disorders, and that this dysconnectivity relates to clinical symptoms and emotion-processing performance (e.g. Peng *et al.* 2014), we hypothesized that VPT individuals would exhibit: (1) reduced rs-fc between the amygdala and key nodes of an emotion-processing network (Leppänen & Nelson, 2009); (2) lower accuracy and longer reaction times than controls at recognizing emotions at lower intensity levels; and (3) functional integrity of the amygdala connectivity network would be related to performance on the ERT. *Post-hoc* exploratory analyses investigating associations between rs-fc, emotion recognition, full-scale IQ and perinatal variables were additionally conducted.

## Method

### Participants

We studied 36 participants recruited from a cohort of 218 individuals who were born at <33 weeks of gestation at University College London Hospital between 1979 and 1984 and who were enrolled into a follow-up study (Nosarti *et al.* 2008, 2014). Exclusion criteria were any history of neurological conditions including meningitis, head injury and cerebral infections.

Thirty-eight age-matched controls were recruited from advertisements in the local press and universities. Inclusion criteria were full-term birth (38–42 weeks of gestation) and birth weight > 2500 g. Exclusion criteria were any history of birth complications (e.g. endotracheal mechanical ventilation), neurological conditions including meningitis, head injury and cerebral infections.

All participants gave informed written consent, were reimbursed for travel expenses and received a nominal remuneration for participation in the study. The study was given ethical approval by the Psychiatry, Nursing and Midwifery Research Ethics Subcommittee, King's College London.

## Materials

IQ was assessed by the Wechsler Abbreviated Scale of Intelligence (WASI; Wechsler, 1999), which contains four subtests (Vocabulary, Similarities, Block Design and Matrix Reasoning) and provides estimates of verbal, performance and full-scale IQ. Processing speed and sustained attention were assessed using respectively correct response reaction times and omission errors from the Conner's Continuous Performance Test II (CCPT-II; Conners, 2000). Mental health at testing was evaluated with the General Health Questionnaire (GHQ-12; Goldberg & Williams, 1988) and Peters’ Delusional Inventory (PDI-21; Peters *et al.* 1999), that assess respectively anxiety/depression symptoms and psychosis proneness.

The ERT was used to evaluate the ability to identify six universal emotions (anger, disgust, fear, happiness, sadness, surprise) by using dynamic stimuli of human faces at different intensities. The stimuli set was developed from colour pictures of four Caucasian actors (two males and two females) in frontal view, who were asked to look neutral and to show a full-blown emotional expression. For each identity, a computer program created intermediate morphed images starting with an inexpressive frame and ending with an emotional expression of different intensities, ranging from 20% to 100% (Montagne *et al.* 2007). The ERT version used in this study included only four levels of intensity: 0–40%, 0–60%, 0–80% and 0–100%.

The presentation procedure started with an instruction screen and a short practice trial with stimuli at 0–100% intensity. The experimental task consisted of a total of 96 stimuli divided into four continuous blocks of increasing intensity. In each trial, one video-clip of a facial expression was played on the screen, the last frame of which represented a static image at the final emotional intensity. Then six labels, describing the six emotions, appeared on the screen and participants were requested to choose the one which was deemed to correspond to the preceding facial expression. No time restriction was imposed, but reaction times were recorded. Overall ERT administration duration was approximately 10 min.

### Socio-demographic, perinatal, neuropsychological and ERT data analysis

Statistical analyses were performed using SPSS v. 22.0 (IBM SPSS Statistics, USA). Group comparisons in terms of age at assessment and neuropsychological test scores were performed using independent-sample *t* tests or their non-parametric equivalent. Sex distribution was tested with Pearson's χ^2^, whereas differences in socioeconomic status (SES) and ethnicity were explored with Fisher's exact test. Differences in neonatal characteristics and full-scale IQ between the VPT group and the larger sample of VPT participants studied at 19–20 years (Nosarti *et al.* 2014) were analysed with univariate analysis of variance (ANOVA).

ERT performance was analysed in terms of both accuracy, as the number of correctly labelled expressions per emotion per intensity (maximum = 4), and mean reaction time for each emotion-intensity level. Both measures were analysed using univariate analysis of covariance (ANCOVA) adjusting for processing speed as defined earlier (CCPT-II correct-response reaction times). First, modulation of accuracy and reaction times were investigated in separate three-way mixed ANCOVAs with emotion type (six levels: anger, disgust, fear, happiness, sadness, surprise) and emotion intensity (four levels: very low 0–40%, low 0–60%, high 0–80%, very high 0–100%) as within-subjects factors and group (two levels: preterm *v.* control) as between-subject factor. Subsequently, a two-way mixed ANCOVA was performed for each emotion separately, with emotion intensity as within-subject factor and group as between-subject factor. Greenhouse–Geisser correction was used for violations of sphericity. False discovery rate (FDR) correction was applied throughout the analyses to account for multiple comparisons.

### MRI data acquisition

Neuroimaging data were acquired at the Maudsley Hospital (London) using a General Electric Signa HDx 3.0 T MR scanner (GE Healthcare, USA). Resting-state images were collected from a gradient-echo echo-planar sequence (TR/TE: 2000/30 ms, flip angle: 75°, matrix 64 × 64, FoV = 218 cm) resulting in 256 whole-brain volumes contained 37 non-contiguous slices with 2.4-mm thickness, 1-mm interslice gap and 3.4-mm voxel resolution. Before the resting-state session, participants were instructed to remain still with gaze fixed on a central cross.

For spatial normalization and localization, high-resolution T1-weighted anatomical images were also acquired using a SPGR pulse sequence with the following parameters: TR = 7.1 ms, TE = 2.8 ms, TI = 450, FOV = 280 cm, flip angle = 20°, matrix = 256 × 256, 196 slices, no interslice gap, slice thickness = 1.1 mm, isotropic resolution = 1.1 × 1.1 × 1.1 mm^3^.

### MRI data pre-processing

Images were processed and analysed in SPM8 (http://www.fil.ion.ucl.ac.uk/spm/software/spm8/) running on Matlab 7.12 (MathWorks, USA). Data were slice-time corrected, spatially realigned, normalized to a sample-specific DARTEL template created on the basis of unified segmentation of individual anatomy (Ashburner, 2007), and smoothed with a 6 mm full-width at half-maximum (FWHM) isotropic Gaussian kernel.

### Head motion and artefact detection

As recent studies have demonstrated resting-state fMRI metrics to be particularly sensitive to confounding effects of head motion (Power *et al.* 2012; Van Dijk *et al.* 2012), the Artifact Detection Tools (ART, www.nitrc.org/projects/artifact_detect/) were used to identify problematic time points. In particular, an image was defined as an outlier (artefact) if a participant's head movement differed by in excess of 0.5 mm (translation) or 0.02 rad (rotation) from the previous frame, or if the global mean intensity in the image was >3 s.d. from the mean image intensity for the entire scan.

To preserve the temporal structure of the data, outlier volumes were not corrected or deleted from the time series, but rather modelled in the first level General Linear Model (GLM). A Mann–Whitney *U* test revealed no significant difference between VPT-born (median = 2.50) and controls (median = 2.00) in the number of outlier volumes (*z* = −0.607, *p* = 0.544).

### Regions of interest (ROIs) definition

Rs-fc MRI data were analysed using a seed-based approach and taking the amygdalar subcompartments (supported by convergent structural and functional observations; Bzdok *et al.* 2013) as ROIs. Three amygdalar seed regions were determined through stereotaxic probabilistic maps of cytoarchitectonic boundaries (Amunts *et al.* 2005), implemented in the SPM Anatomy toolbox (Eickhoff *et al.* 2005). The centromedial group included the central and medial nuclei; the laterobasal subregion consisted of the lateral, basolateral, basomedial, and paralaminar nuclei; the superficial group included the anterior amygdaloid area, the amygdalopyriform transition area, the amygdaloid-hippocampal area and the ventral and posterior cortical nuclei.

Only voxels with at least a 50% probability of belonging to one of these subregions were included, and those exceeding 50% probability for multiple subregions were assigned only to the region for which they had the highest probability of inclusion. Images of each subregion were created in the standard space (MNI) separately for left and right hemisphere to create a total of six ROIs.

### Rs-fc MRI seed-region analysis

The Functional Connectivity toolbox (CONN v14.b) (Whitfield-Gabrieli & Nieto-Castanon, 2012; http://web.mit.edu/swg/software.htm) was used to perform all seed-based analyses. To remove physiological noise and movement confounds, the CONN toolbox uses the anatomical component correction (aCompCor) strategy (Behzadi *et al.* 2007) that increases the sensitivity and specificity of positive correlations and can detect non-artifactual anti-correlations. Global signal regression was excluded to avoid the introduction of artificial negative correlation (Murphy *et al.* 2009). Subject-specific effects of motion (defined by six realignment parameters and their first-order temporal derivatives), outliers volumes (represented by a binary regressor), and white matter and CSF signals (characterized by the first five principal components of each) were removed from the functional data using linear regression and the resulting residual blood oxygen-level dependent (BOLD) time series were band-pass filtered between 0.009 and 0.08 Hz.

In the first-level analyses, the average BOLD time-course was extracted from each seed region separately and Pearson's correlation coefficients were computed between that time course and the time course of every other voxel in the brain. Seed-to-voxel connectivity maps were constructed for each subject. Correlation coefficients were then converted to normally distributed *z* scores using the Fisher transformation to allow the second-level GLM analyses. Within-group statistical parametric maps (SPMs) and connectivity differences between groups were further explored and examined with independent-sample *t* tests. Reported clusters survived a height threshold of uncorrected *p* < 0.001 and an extent threshold of FDR-corrected *p* < 0.05 at cluster level.

### Associations between rs-fc MRI and ERT, socio-demographic, neuropsychological and perinatal data

To evaluate relationships between rs-fc MRI data and emotion-processing performance, *post-hoc* correlation analyses were conducted. In these analyses, correlations between connectivity of significant clusters obtained during second-level analyses and ERT emotion recognition accuracy were assessed with Spearman's rank test.

Multiple analyses of variance were used to test for the effects of sex and SES, as well as interactions with group, in terms of ERT metrics and rs-fc patterns found to be different between preterm and control participants.

Other associations between rs-fc, full-scale IQ and perinatal factors were assessed using Pearson's correlation coefficient for parametric continuous variables.

## Results

### Sample characteristics

Characteristics of study sample are reported in Table 1. Controls tended to belong to higher SES compared to VPT individuals and about one quarter (26%) of controls was recruited from the university campus (three post-doctoral researchers, three research workers/technicians, four postgraduate students). In terms of neuropsychological and mental health outcomes, compared to controls, VPT participants had significantly lower verbal, performance and full-scale IQ, despite their values falling within the test norm. No statistically significant between group differences were found in processing speed, sustained attention, or current mental health (GHQ-12 and PDI-21).

**Table 1.** Sample characteristics. Frequencies, percentages and mean values (standard deviations) are given, unless otherwise specified

	VPT (*N* = 36)	Controls (*N* = 38)	Statistics
Demographic and neonatal characteristics			
Sex (male/female)	20/16	17/21	χ^2^_1_ = 0.87, *p* = 0.352
Age at assessment (years)	30.25 (2.31)	29.16 (3.64)	*t*_72_ = 1.53, *p* = 0.130
Birth weight (g)	1295.44 (358.93)	n.a.	n.a.
Gestational age (weeks)	29.08 (2.34)	n.a.	n.a.
Socioeconomic status^a^			
I–II	47.2%	71.1%	Fisher's exact test = 10.47, *p* = 0.020
III	25%	15.8%	
IV–V	8.3%	0%	
Student	2.8%	10.5%	
Unemployed/out of work	16.7%	2.6 %	
Ethnicity			
Caucasian	80.6%	84.2%	Fisher's exact test = 2.89, *p* = 0.647
African	0%	2.6%	
Afro-Caribbean	0%	2.6%	
Indian subcontinent	11.1%	5.3%	
Other	8.3%	5.3%	
Neonatal US classification^b^			
Normal	17	n.a.	n.a.
Uncomplicated PVH	9	n.a.	n.a.
PVH + DIL	10	n.a.	n.a.
Neuropsychological assessment			
Full-scale IQ	103.94 (14.08)	113.68 (10.62)	*t*_72_ = 3.37, *p* = 0.001
Verbal IQ	100.72 (15.01)	110.66 (12.51)	*t*_72_ = 3.01, *p* = 0.003
Performance IQ	106.33 (14.17)	114.00 (9.34)	*t*_72_ = 2.76, *p* = 0.008
Processing speed	421.67 (58.53)	415.26 (57.82)	*t*_72_ = 0.47, *p* = 0.637
Sustained attention^c^	1.50 (1.64–6.36)	2.00 (2.12–4.25)	*U* = 607.00, *p* = 0.399
Mental health assessment			
GHQ-12^c^	2.00 (2.04–4.51)	1.50 (1.21–3.42)	*U* = 558.00, *p* = 0.160
PDI-21^c^	22.50 (22.92–45.80)	10.00 (13.58–32.37)	*U* = 535.50, *p* = 0.107

VPT, Very preterm birth.

a Socioeconomic status classified according to the Standard Occupational Classification 1980 (SOC1980). The following categories were used: I−II = managerial and professional; III = intermediate (e.g. small employers and own account workers); IV−V = working (e.g. lower supervisory and technical occupations, routine and semi-routine occupations).

b Periventricular haemorrhage was classified as haemorrhage into the germinal layer or lateral ventricles and ventricular dilatation as clear dilatation of one or both lateral ventricles with cerebrospinal fluid, although not sufficient to meet the conditions for a diagnosis of hydrocephalus (Stewart *et al.* 1983).

c Median and 95% confidence intervals.

In order to assess selection bias in the VPT sample, we compared demographic and perinatal characteristics between the current VPT sample and a sample of 68 individuals who were assessed at age 19–20 (Nosarti *et al.* 2014). There were no statistically significant differences between the two VPT cohorts in terms of gestational age (*F*_1,102_ = 0.24, *p* > 0.05) and birth weight (*F*_1,102_ = 0.85, *p* > 0.05), although the current cohort had higher full-scale IQ than the VPT cohort assessed at age 19–20 (*F*_1,102_ = 7.29, *p* < 0.01).

### Emotion Recognition Task

**Fig. 1.**
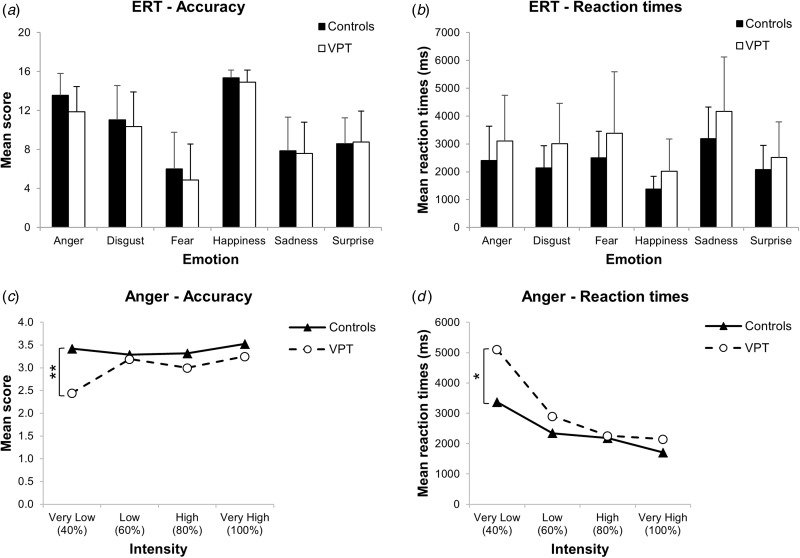
Emotion Recognition Task (ERT) performance. (*a*) Mean scores and (*b*) mean reaction times of very preterm birth (VPT) participants and controls for the six ERT emotions. Error bars represent 1 standard deviation. (*c*) Mean scores and (*d*) mean reaction times of VPT participants (white dots) and controls (black triangles) at the four levels of intensity for anger. *p < 0.05, **p < 0.001.

Preterm participants’ and controls’ performance on the ERT in terms of *accuracy* and *reaction time* are displayed in Fig. 1 (*a*, *b*).

Regarding *accuracy*, a three-way mixed ANCOVA demonstrated a trend towards a significant main effect for group (*F*_1,71_ = 3.13, *p* = 0.081), with controls tending to perform better than VPT adults. Interactions between group and emotion type (*F*_5,355_ = 1.11, *p* = 0.352) and emotion intensity (*F*_3,213_ = 1.84, *p* = 0.149) were not significant. When analysing each emotion separately, a significant main effect for group (*F*_1,71_ = 9.05, *p* = 0.024) and the interaction between group and emotion intensity (*F*_3,213_ = 5.86, *p* = 0.006) were found only for anger. Further ANCOVAs showed that VPT adults were less able than controls to recognize anger at the lowest level of intensity; i.e. the most difficult condition (*F*_1,71_ = 23.41, *p* < 0.0001; Fig. 1*c*).

Considering *reaction time*, a three-way mixed ANCOVA demonstrated a significant main effect for group (*F*_1,71_ = 8.48, *p* = 0.005), with controls being faster than VPT participants. There was also a significant interaction between group and emotion intensity (*F*_3,213_ = 7.01, *p* = 0.005), showing that controls were faster than VPT participants at each level of intensity, with reaction times progressively decreasing as the emotion became more obvious (e.g. at higher levels of intensity). When analysing each emotion separately, as it was done for accuracy, there was a significant interaction between emotion intensity and group only for anger, after FDR-correction (*F*_3,213_ = 5.55, *p* = 0.042). Further ANCOVAs showed that VPT adults were slower than controls at recognizing anger at the lowest level of intensity (*F*_1,71_ = 7.15, *p* = 0.036; Fig. 1*d*).

### Rs-fc MRI results

#### Within group rs-fc MRI results

Within-group connectivity maps and significant areas of activation across the six amygdalar subregions (three in each hemisphere) are presented in Supplementary Fig. S1. In brief, spontaneous activity in the amygdala subregions was positively associated with neuronal activity in surrounding structures and the ventromedial prefrontal cortex, whereas it was negatively associated with neuronal activity in dorsal prefrontal cortex, parietal lobe and posterior brain regions, with strong inter-hemispheric similarities.

#### Between-group rs-fc MRI results

**Fig. 2.**
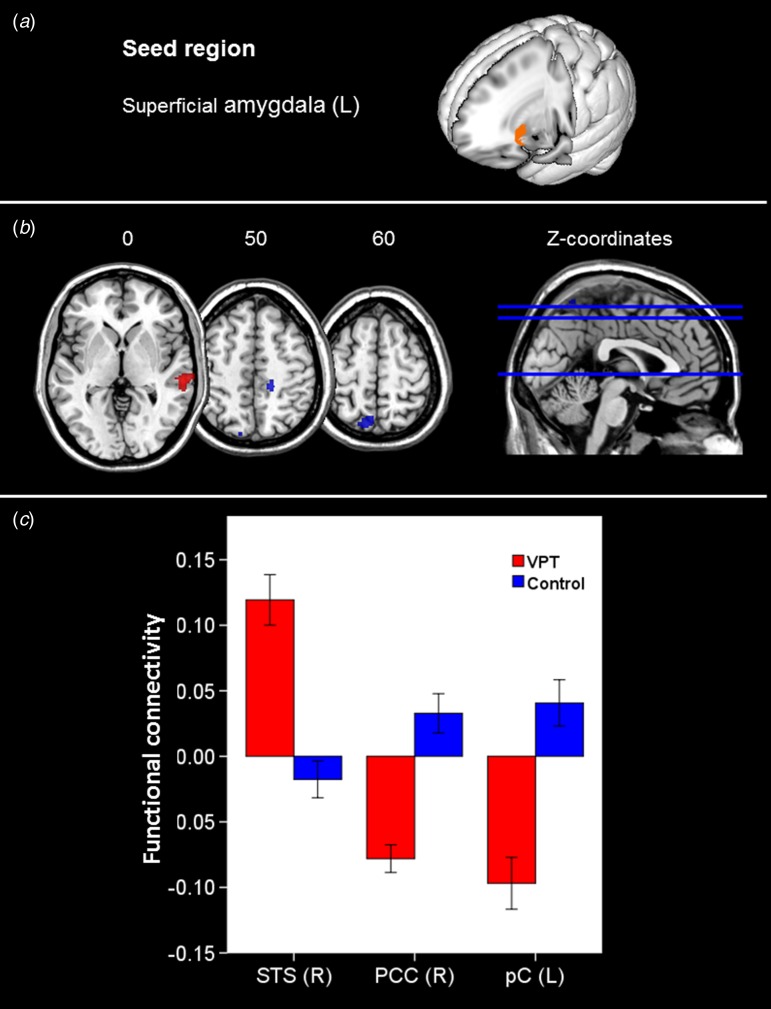
Alterations in functional connectivity of the left superficial amygdala in very preterm birth (VPT)  individuals compared to controls. (*a*) Location of 50% probabilistic masks of the superficial amygdala of the left hemisphere projected on the MNI template with neurological convention. (*b*) Brain areas that showed significantly greater functional connectivity of the left superficial amygdala in VPT individuals (hot colour scale) comprised the right superior temporal sulcus (STS); brain areas that showed significantly smaller functional connectivity of the left superficial amygdala in VPT individuals (cool colour scale) comprised the right posterior cingulate cortex (PCC) and the left precuneus (pC). Brain slices are displayed axially according to neurological convention, with z coordinates in MNI space above each slice. The figure on the right depicts these slices in blue on a midline, sagittal slice. Reported clusters survived a height threshold of uncorrected *p* < 0.001 and an extent threshold of FDR-corrected *p* < 0.05 at the cluster level. (*c*) Bar graph showing direct comparison of functional connectivity strength between the two groups. The y-axis indicates correlation coefficients between the time series of the seed region (superficial subregion of the left amygdala) and the time series extracted from the regions displaying significant between-group differences; the error bars represent 1 standard error.

Significant between-group differences in patterns of rs-fc were found only for connectivity of the left superficial amygdalar subregion. This area showed hypoconnectivity with the right posterior cingulate cortex (PCC) and the left precuneus (pC) and hyperconnectivity with the superior temporal sulcus (STS) in VPT adults compared to controls (Table 2, Fig. 2).

**Table 2.** Differences in functional connectivity of the left superficial amygdala between VPT individuals and controls

Contrast	Location (hemisphere)	Cluster size	Cluster *p* value	MNI coordinates			Peak voxel *t* statistic
				x	y	z	
VPT < Controls	Cingulate gyrus (R)	148	0.017	20	−26	44	5.13
				16	−34	48	5.07
				22	−16	44	3.83
				16	−16	42	3.48
	Precuneus (L)	238	0.002	−10	−70	60	4.29
				−14	−76	44	3.85
VPT > Controls	Superior temporal gyrus (R)	166	0.008	62	−28	0	5.15

VPT, Very preterm birth; R, right hemisphere; L, left hemisphere.

Reported clusters survived a height threshold of uncorrected *p* < 0.001 and an extent threshold of FDR-corrected *p* < 0.05 at the cluster level.

### Investigating associations between rs-fc MRI and facial emotion processing, sex, SES, IQ and perinatal risk factors

**Fig. 3.**
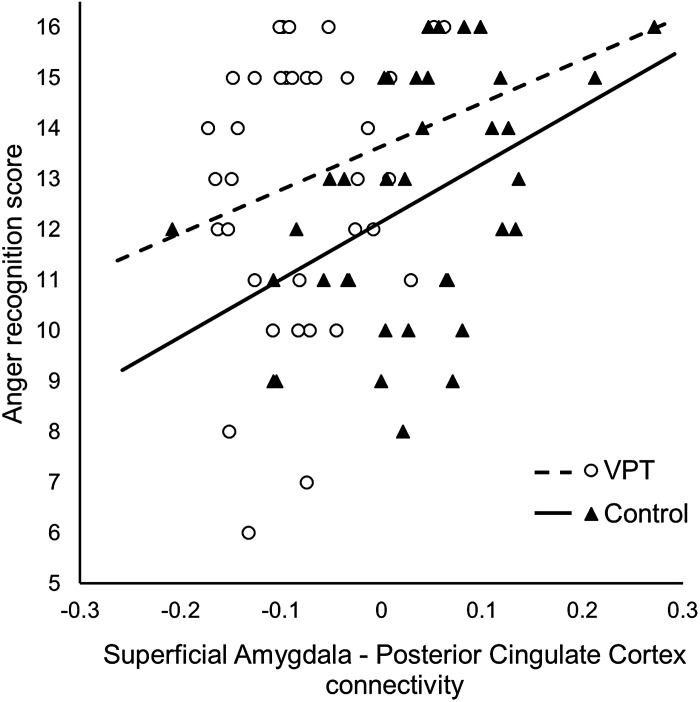
Correlation between superficial amygdala-posterior cingulate connectivity and Emotion Recognition Task score for anger in very preterm birth (VPT) participants (white dots; Spearman's *ρ* = 0.172) and controls (black triangles; Spearman's *ρ* = 0.426). Least-squared lines are shown for illustrative purposes only and do not constitute a formal test.

In controls, anger scores were significantly associated with rs-fc between the left superficial amygdala and the PCC (Spearman's *ρ* = 0.426, *p* = 0.008), whereas in the VPT group this association was not significant (Spearman's *ρ* = 0.172, *p* = 0.316; Fig. 3). However, the correlation was not significantly different between the groups (*z* = 1.16, *p* = 0.246).

*Post-hoc* analyses to assess the effect of sex and SES demonstrated no statistically significant differences between males and females, nor between SES categories on rs-fc patterns and ERT emotions where differences between preterm participants and controls were detected (all *p* > 0.05).

Analysis of the association between rs-fc and full-scale IQ in VPT participants and controls did not show any statistically significant result (see Supplementary Table S1).

Within the VPT participants, perinatal risk factors (e.g. gestational age, birth weight and neonatal ultrasound classification) did not significantly correlate with either ERT performance or rs-fc patterns.

## Discussion

Several investigations have suggested an association between atypical social development and VPT. This study provides two important contributions that help to elucidate the mechanisms underlying a possible link between the two. First, our results suggest that in adulthood VPT individuals display functional alterations in brain circuits fundamental to emotion processing; second, they demonstrate that VPT adults are worse than controls at recognizing subtle specific emotions from facial expressions.

Recognizing facial expressions denoting specific emotions is a crucial skill for successful interpersonal interactions and is compromised in many psychiatric disorders with onset in childhood/adolescence (Law Smith *et al.* 2010; Jarros *et al.* 2012; Collin *et al.* 2013) and adulthood (Scholten *et al.* 2005; Montagne *et al.* 2006, 2008; Poljac *et al.* 2011). In this study, findings from the overall ERT demonstrated that, compared to controls, VPT-born individuals had almost intact ability, but longer reaction times, at recognizing emotion expressions. This could be interpreted as a compensatory mechanism in VPT adults, who need more time to adequately process emotional stimuli. A more detailed analysis of emotion type found specific and subtle impairments in VPT adults in recognizing anger at very low intensity, with their performance being both slower and less accurate than controls. Importantly, in the current study these results are unlikely to depend on VPT participants’ deficits in attention/processing speed or psychological distress, as their scores on such measures were comparable to controls’. These findings suggest that deficits in emotion recognition – previously reported generically at the age of ⩽5 years (Potharst *et al.* 2013; Witt *et al.* 2014) and specifically for anger and low-intensity stimuli at 8 years (Wocadlo & Rieger, 2006) – persist into adulthood in VPT-born individuals. In the literature, a selective impairment in recognizing angry expressions has been associated with lower social competence in children across different ages (Maxim & Nowicki, 2003). Diminished social competence could further result in social isolation and rejection by peers, as reported in VPT adolescent samples (Rickards *et al.* 2001; Dahl *et al.* 2006). Additionally, a reduced ability to recognize a potential threat conveyed by angry faces may make children more vulnerable to being bullied by peers (Woods *et al.* 2009), placing VPT samples at risk of experiencing bullying and related emotional problems (Wolke *et al.* 2015).

Even though these results suggest a selective difficulty for anger, the null findings in other emotions are likely to be due to a small sample size and the fact that the current VPT participants were cognitively unimpaired individuals (i.e. had IQ scores within the test norm). The possibility that VPT participants would also show subtle deficits for other emotions cannot be excluded by the current results and warrants testing in a larger sample with more variable psychosocial functioning. Moreover, a precise measure of participants’ mood at testing would potentially mitigate potential bias effects on emotion processing.

To investigate the neural underpinnings of the observed behavioural deficits, whole-brain rs-fc analyses were conducted using seeds in three cytoarchitectonic subdivisions of the amygdala (and thus permitting dissociation of regionally specific connectivity patterns). Indeed, the superficial subregion of the left amygdala was unique in showing evidence of altered rs-fc in VPT participants compared to controls. While control subjects exhibited significant positive connectivity between this region and pC as well as PCC, VPT-born individuals displayed significant negative functional connectivity between the same regions. VPT-born participants also showed increased connectivity between left superficial amygdala and the STS. Functional aberrations specific to this amygdalar compartment are not unexpected in light of recent meta-analytical findings (Bzdok *et al.* 2013) which reported that the superficial amygdala is highly tuned for processing both static and dynamic social stimuli in the form of emotional expression of human faces (Hurlemann *et al.* 2008; Goossens *et al.* 2009), and structural alterations of this subregion have been described in anxiety disorder (Qin *et al.* 2014).

Consistent with previous findings from healthy subjects (Roy *et al.* 2009), both groups in the current study displayed negative correlations between activity in amygdala and dorsolateral prefrontal and posterior parietal regions typically involved in cognitive control of emotions. However, VPT-born individuals displayed a wider suppression of posterior areas, which extended to a larger proportion of the PCC and the bilateral pC. Aberrant amygdala–PCC/pC coupling has been previously reported in psychiatric samples with reduced connectivity associated with state anxiety scores (Hahn *et al.* 2011) and elevated connectivity found in individuals diagnosed with major depressive disorder (Cullen *et al.* 2014). The PCC and pC are involved in emotional evaluation (Wright *et al.* 2008) and modulation (Adolphs, 2003), and also constitute core regions of the ‘default mode network’ (DMN), a system that is more active at rest than during goal-oriented tasks (Raichle *et al.* 2001; Greicius *et al.* 2003). Increasing evidence demonstrates a substantial overlap between the DMN and the ‘social brain’ (Mars *et al.* 2012) and a relationship of reciprocal inhibition has been proposed between the cognitive and emotional systems (Drevets & Raichle, 1998). We previously showed alterations in rs-fc between posterior DMN and salience network in VPT adults from the same cohort compared to controls (White *et al.* 2014). In this framework, hypoconnectivity between the amygdala and the PCC/pC in VPT-born individuals could hint at an altered coupling of these two regions. It is feasible that amygdalar activation in socio-emotional relevant conditions might excessively suppress the activity of DMN brain regions responsible for cognitive evaluation of a given situation. This interpretation is in line with the study by Sreenivas *et al.* (2012), which reported a deactivation of DMN regions during the processing of emotional faces and, in particular, a greater deactivation for negative compared with positive emotions, as a demonstration of their higher evolutionary saliency. An increased negative coupling between the amygdala and the PCC/pC could represent an overactive inhibitory connection that could become disruptive and maladaptive, not only in ‘spectatorial’ and experimental tasks, but also in emotionally-engaged and interactive settings in real life (Schilbach *et al.* 2013), as there exists a large topological overlap of brain regions involving such processes.

The current study suggests that the pathway between PCC and amygdala importantly contributes to emotion recognition. Connectivity between PCC and the left superficial amygdala was significantly associated with the ability to recognize angry faces in controls but not in VPT individuals, and suggests this as a potential substrate for selective ERT impairments in the latter. As the dorsal PCC plays a key modulatory role in the amygdala network for emotion processing (Pessoa *et al.* 2005; Stein *et al.* 2007), it can be reasonably hypothesized that the inability to recognize low intensity levels of anger may result from inadequate amygdalar modulation in VPT-born individuals. This has potential implications for social cognition, especially in situations where it is necessary to attribute mental states to others (Saxe & Powell, 2006). The PCC is a highly connected structure (Hagmann *et al.* 2008) responsible for self-referential thought (Johnson *et al.* 2002; Mason *et al.* 2007; Buckner *et al.* 2008; Whitfield-Gabrieli *et al.* 2011), but is also involved in cognitive processes (Pearson *et al.* 2011). It acts as a connector hub in cortico-subcortical networks (Leech *et al.* 2012); and has been found to influence activity in distributed networks responsible for performing goal-directed actions, such as the dorsal attention network, fronto-parietal control network and salience network (Leech & Sharp, 2014). In particular, the dorsal PCC is involved in allocating attentional focus and balancing between internally- and externally-oriented cognition (Leech *et al.* 2011, 2012). Thus, socio-emotional deficits in VPT-born individuals may result from difficulties in coordinating activity between systems responsible for processing emotion and those responsible for attention modulation. Impairments in executive function are widely recognized in VPT-born individuals across the life span (Aarnoudse-Moens *et al.* 2009; Burnett *et al.* 2013; Sølsnes *et al.* 2014) and these have been hypothesized as a potential mechanism to understand socio-emotional difficulties in VPT children (Hille *et al.* 2001). In other words, difficulties with adaptability, impulsivity, and attention could affect social interactions. Although our current analyses showed significant between group differences in ERT performance after accounting for sustained attention, it remains to be investigated whether impaired modulation of different aspects of attention (e.g. attentional control) which were not measured in the current study affect emotion recognition in VPT samples (Eack *et al.* 2016).

This study additionally presents evidence of connective pathways supporting emotional processing that are specific to individuals born VPT. Most notably, increased connectivity was found between the left superficial amygdala and the STS in this group but not in controls. Structural alterations in the STS have been previously reported in VPT samples (Nosarti *et al.* 2008; Rogers *et al.* 2014). This region – and in particular its posterior portion (Hein & Knight, 2008)- is well known as a face-sensitive region specialized for dynamic feature recognition (Haxby *et al.* 2000; Andrews & Ewbank, 2004; Engell & Haxby, 2007), including eye and mouth movements (Puce *et al.* 1998; Hoffman & Haxby, 2000) and emotional expressions (LaBar *et al.* 2003). Together with the amygdala and the orbitofrontal cortex, the STS has been proposed as a key region in social cognition (Brothers, 1990; Allison *et al.* 2000). Decreased connectivity between the amygdala and the STS has been hypothesized as the underlying mechanism of emotion and face processing disturbance in various psychiatric disorders, including autism spectrum disorder (Monk *et al.* 2010) and major depressive disorder (Ramasubbu *et al.* 2014). By contrast, the increased amygdala–STS connectivity found in our VPT-born sample could represent a compensatory strategy in a suboptimally efficient emotional network, facilitating emotion recognition by means of stronger integration between decoding of facial characteristics by the temporal areas and emotional salience by the amygdala. This interpretation could also explain the overall slower and less accurate performance of VPT adults on the ERT. Moreover, as the STS is crucial for theory of mind (Gallagher & Frith, 2003; Vander Wyk *et al.* 2009), hyperconnectivity of this region with the amygdala could also support higher cognitive processing of emotional information.

### Strengths, limitations and future directions

There are several limitations in this study. First, the amygdala, which was chosen as a seed region for rs-fc analyses, is susceptible to EPI image distortion and signal dropout, which may cause relevant problems of spatial localization (Merboldt *et al.* 2001). To deal with this potential bias, only the core of each amygdalar subregion was considered by using a probabilistic map at 50%, as in Roy *et al.* 2009, but a stricter threshold could have been used (e.g. 90%, as in Ball *et al.* 2007). Second, a score range between 0 and 4 at each ERT level of emotion did not allow us to explore subtler impairments in emotion recognition in relation to altered patterns of amygdalar connectivity. An increased number of trials or the use of more numerous levels of emotional intensity to further aggregate could have refined our analyses. Finally, the fact that controls tended to belong to higher SES bands compared to VPT individuals could have influenced our findings, although the current sample included high performing VPT adults (reflected by their higher IQ scores compared to a larger VPT sample assessed at age 18–19 years), who nevertheless had significantly lower IQ than controls. Replication of the study with a larger sample size might address these issues and provide a means of uncovering additional subtle features on account of increase statistical power.

Strengths of our study include: (1) a choice of amygdalar subdivisions used to define seed regions for rs-fc analysis, that permitted the investigation of between-group differences that could not have been detected considering the whole amygdala; (2) the use of DARTEL toolbox, that achieved a more accurate registration and normalization to account for structural differences expected between VPT-born individuals and born-at-term controls; and (3) the use of the ERT, which contains dynamic facial information, rather than fully blown and/or static information, which has proved advantageous in terms of identification of emotional expressions, evaluations of intensity and arousal, and discrimination of authenticity (Krumhuber *et al.* 2013), making it a useful tool to detect subtle and selective impairment that previous research failed to find (e.g. Ogai *et al.* 2003).

Future studies in VPT samples could investigate other critical skills that rely on emotion perception and contribute to the understanding of the complexity of the social world, such as attributing mental states, deciphering emotional meanings, using emotions in thought, and managing emotions (Mayer *et al.* 2001). These components could differentially contribute to behavioural and psychiatric problems and they are still unexplored in VPT populations. Potential interventions that aim at improving socio-emotional skills could then strengthen different components of emotional intelligence (e.g. Bölte *et al.* 2015).

## Conclusion

VPT individuals continue to display subtle emotion recognition deficits in adulthood, which are associated with functional alterations in brain circuits fundamental to emotion processing, characterized by suboptimal amygdala modulation. These findings provide a potential foundation for future studies investigating how emotion-processing impairments may interact with biological, environmental and genetic risk and contribute to the increased vulnerability to psychiatric disorder in VPT individuals.

## Supplementary material

For supplementary material accompanying this paper visit http://dx.doi.org/10.1017/S0033291716001604. click here to view supplementary material
