# Supplementary material for: Altered resting-state functional connectivity in emotion-processing brain regions in adults who were born very preterm
Source: Psychol Med. 2016 Aug 15;46(14):3025–39. doi: 10.1017/S0033291716001604 (PMC5080670; doi:10.1017/S0033291716001604)
Supplement: Supplementary file 1 [file S0033291716001604sup001.zip › FigureS1.docx]

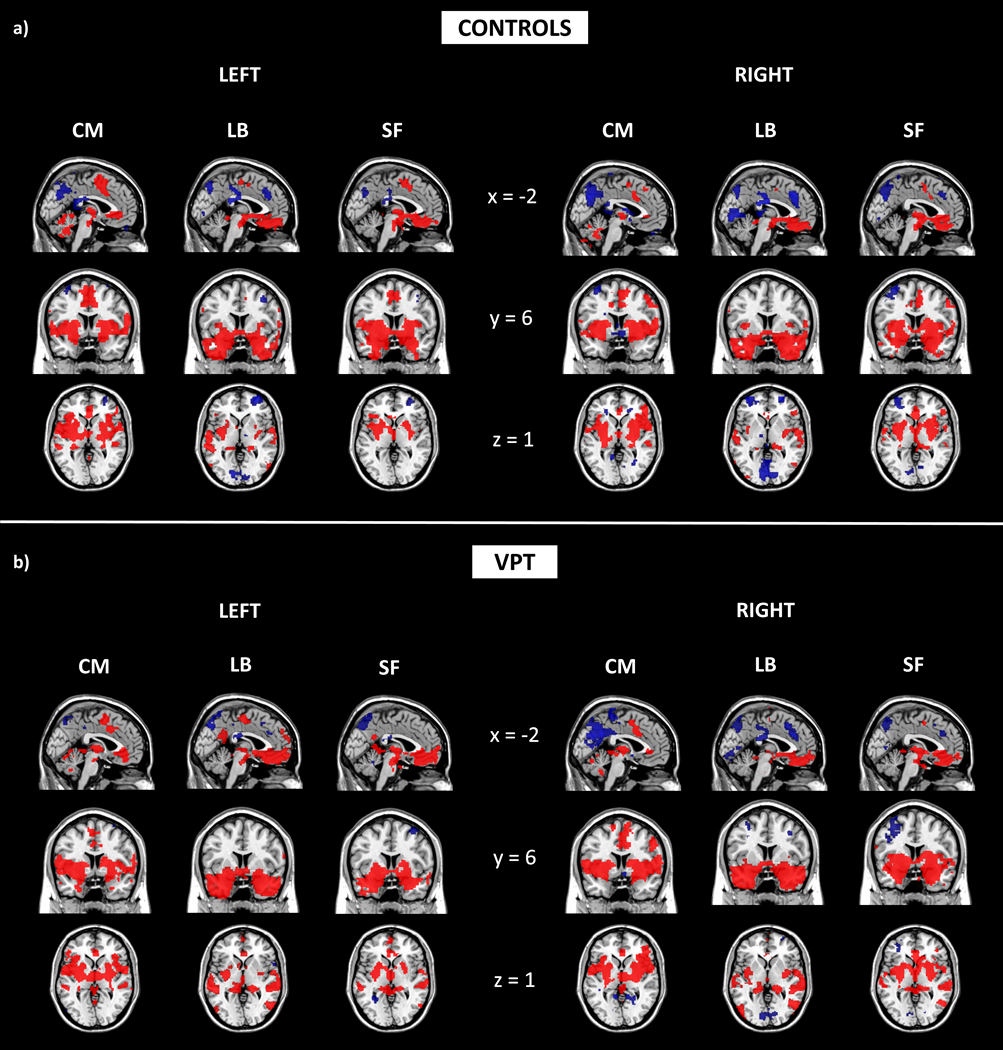


**Supplementary Fig. S1.** Within-group connectivity maps of the amygdala for controls (a) and VPT-born individuals (b). Red and blue represent respectively positive and negative relationships for the laterobasal (LB), centromedial (CM) and superficial (SF) subregions. All results are overlaid on a standardized T1-weighted template brain and presented in sagittal (x = -2), coronal (y = 6) and axial (z = 1) views (neurological convention). Reported clusters survived a height threshold of uncorrected *p* < .001 and an extent threshold of FDR-corrected *p* < .05 at the cluster level.
