# Supplementary material for: Altered resting-state functional connectivity in emotion-processing brain regions in adults who were born very preterm
Source: Psychol Med. 2016 Aug 15;46(14):3025–39. doi: 10.1017/S0033291716001604 (PMC5080670; doi:10.1017/S0033291716001604)
Supplement: Supplementary file 1 [file S0033291716001604sup001.zip › TableS1.docx]

**Supplementary Table S1.** *Correlation coefficients between IQ and the amygdala resting-state functional connectivity in VPT born participants and controls (after FDR correction).*

|  | STS (R) | | PCC (R) | | pC (L) | |
| --- | --- | --- | --- | --- | --- | --- |
|  | *r* | *p* | *r* | *p* | *r* | *p* |
| VPT | -0.133 | 0.995 | -0.075 | 0.995 | 0.010 | 0.955 |
| Controls | -0.353 | 0.090 | -0.058 | 0.728 | -0.122 | 0.696 |

L = left

PCC = posterior cingulate cortex

PC = precuneus

R = right

STS = superior temporal sulcus
